# A trained communication partner’s use of responsive strategies in aided communication with three adults with Rett syndrome: A case report

**DOI:** 10.3389/fpsyg.2022.989319

**Published:** 2022-09-29

**Authors:** Helena Wandin, Per Lindberg, Karin Sonnander

**Affiliations:** ^1^Disability and Habilitation, Department of Public Health and Caring Sciences, Uppsala University, Uppsala, Sweden; ^2^National Center for Rett Syndrome and Related Disorders, Frösön, Sweden; ^3^Clinical Psychology, Department of Psychology, Uppsala University, Uppsala, Sweden

**Keywords:** responsive strategies, intervention, augmentative and alternative communication (AAC), gaze-controlled device, responsive augmentative and alternative communicative style scale (RAACS), Rett syndrome

## Abstract

**Purpose:**

To explore and describe a trained communication partner’s use of responsive strategies in dyadic interaction with adults with Rett syndrome.

**Introduction:**

Responsive partner strategies facilitate social, communicative, and linguistic development. The common feature is that the communication partner responds contingently to the other’s focus of attention and interprets their acts as communicative. Research on responsive partner strategies that involves individuals with significant communication and motor disabilities remains sparse. The same applies to if, and how, the use of communication aids impacts on the partner’s use of responsive strategies.

**Materials and methods:**

A therapist, trained in responsive partner strategies and aided communication interacted during 14 sessions with each of three participants. The participants were adults with Rett syndrome. A gaze-controlled device and responsive strategies were used during all sessions. The Responsive Augmentative and Alternative Communication Style scale (RAACS) was used to assess the partner’s responsiveness. RAACS consists of 11 items including ratings of to what extent the partner is being attentive to, confirms, and expands the individual’s communication. During eight of the 14 sessions, aided AAC Modelling was also used, i.e., the communication partner pointed at symbols on the gaze-controlled device while interacting. In addition to RAACS, each time the communication partner confirmed or expanded on communication when (a) the participants used the gaze-controlled device and (b) the participants did not use the gaze-controlled device was counted. Descriptive statistics were used to present the results. Non-parametric tests were used to compare means between the two conditions and between participants.

**Results:**

Inter-rater agreement for the different RAACS items ranged from 0.73 to 0.96 and was thus found to be fair to excellent. The communication partner’s use of responsive strategies varied when communicating with different participants and the scores were higher when aided AAC modeling was used. The communication partner’s number of responses and use of responsive strategies were higher when the participants communicated through a gaze-controlled device.

**Conclusion:**

The communication partner’s use of responsive and scaffolding strategies is not a fixed construct but varies in interactions with different non-speaking persons. The same is true whether the non-speaking person uses a gaze-controlled device with digitized speech or not.

## Introduction

Individuals with significant motor and communication disabilities often depend on the communication partner’s ability to support the communication. Responsive partner strategies are a set of communication partner behaviors that facilitate social, communicative, and linguistic development for individuals with and without disabilities (Haebig et al., 2013; Karaaslan and Mahoney, 2015; Van Keer et al., 2020). Importantly, these strategies can increase non-speaking individuals’ engagement in and attention to interaction and thus optimize current interactions (Van Keer et al., 2020).

Common features of responsive partner strategies are that the communication partner responds contingently to the other person’s focus of attention, interprets their acts as communicative, and responds accordingly (Mahoney, 2008; Broberg et al., 2012). There are some indications that responsive partner strategies may have a larger impact on individuals who communicate pre-symbolically (Siller et al., 2013). In these interactions, the communication partner relies on interpreting the individual’s potentially communicative acts such as facial expressions, vocalizations, or body movements (Sigafoos et al., 2000; Dhondt et al., 2020).

To develop communication for individuals with significant motor and communication disabilities, responsive partner strategies may be combined with aided alternative and augmentative communication (AAC; Broberg et al., 2012; Wandin et al., 2015; Medeiros and Cress, 2016; Rensfeldt Flink et al., 2022). In aided AAC external tools such as objects, graphic symbols, and/or electronic devices are used to assist communication and communication development. Gaze-controlled computers allow individuals who have limited hand function to access screen activities and to use graphic symbols by focusing their gaze and holding it for a certain amount of time (i.e., dwell time), by blinking, or by using an external switch (Borgestig et al., 2016; Hsieh et al., 2021; Karlsson et al., 2021).

Strategies such as aided AAC modeling (Biggs et al., 2018; O’Neill et al., 2018) and labeling and expanding on the individual’s actions and utterances are used to support aided language and communication development (O’Neill et al., 2018). In aided AAC modeling, the communication partner points at symbols while speaking, with the purpose of providing models of language and communication. The number of studies supporting this approach is increasing (Allen et al., 2017; Biggs et al., 2018; O’Neill et al., 2018). Labeling (or linguistic mapping) refers to when the communication partners put into words, their interpretation of the individual’s actions (Haebig et al., 2013; Bang et al., 2020). Expansions are when the communication partner labels an action or repeats an utterance at a slightly more advanced communicative or linguistic level (Haebig et al., 2013; Bang et al., 2020). Recasts are expansions on spoken or aided symbolic communication (Soto et al., 2020).

Most studies of responsive partner strategies concern parents and their children while fewer studies have involved other communication partners. However, there are indications that teacher responsivity may facilitate engagement and language development in pupils with and without disabilities (McCathren, 2000; Cabell et al., 2011; Shalev and Hetzroni, 2020). Additionally, few studies involve responsive strategies in interaction with adults. Earlier research indicates a need for including care staff in interventions aimed at improving the responsivity of the communication partners (Hostyn and Maes, 2009; Penne et al., 2012).

It is unclear whether contextual and personal factors influence the use of responsive partner strategies with individuals with disabilities. Activities appear to have little influence on the use of responsive partner strategies in dyadic, parental interaction. Medeiros and Cress (2016) did not find any differences in responsiveness when parents and their children played with familiar or unfamiliar toys, and they found no difference when single message speech-generating devices were used or not. This was also the finding in a study that examined parents’ use of responsive partner strategies in different play activities (Andersson and Eriksson, 2019). However, Shalev and Hetzroni (2020) found that responsivity in school staff was higher when they interacted with students with intellectual and developmental disabilities in dyads compared to when they interacted with the students in group sessions. Regarding personal factors, staff with a more positive attitude towards persons with disabilities were more responsive. Their responsiveness was also higher when the staff was more familiar with the students or had more experience of working with students with intellectual disabilities. Other personal factors such as burnout, job satisfaction, and self-efficacy were not correlated with responsiveness in school staff (Shalev and Hetzroni, 2020).

Few studies have specifically explored responsive partner strategies in interactions between a communication partner and individuals with significant communication and motor challenges (Cress et al., 2013; Van Keer et al., 2017, 2020). Rett syndrome is a rare neurodevelopmental disorder causing significant communication and motor impairment and need for support with most daily activities (Neul et al., 2010; Sandweiss et al., 2020). Through technological advances and systematic symbol-based communication interventions, individuals with Rett syndrome can learn to communicate through symbol-based AAC (Grether, 2015; Vessoyan et al., 2018; Townend et al., 2020). Nonetheless, many do not yet master symbol-based communication, which places high demands on the communication partner to support communication (Julien et al., 2014).

Van Keer et al. (2017) showed that the parents of individuals with significant communication and motor disabilities were generally responsive to their children’s behaviors, and their interactions were characterized by warmth, acceptance, and enjoyment. However, the parents in the study group less frequently showed behaviors that encouraged sensorimotor or cognitive achievements than parents of typically developing children matched for gender, age, and educational level. Rensfeldt Flink et al. (2022) examined reports of parents of children with profound intellectual and multiple disabilities after being trained in strategies to promote communication in everyday activities and routines, including responsive partner strategies. The results showed that parents were generally positive to the training, reporting that they consequently gave the child more time to communicate and were more attentive to their children’s communicative signals.

The overall aim of the current study was to explore and describe in detail a trained communication partner’s use of responsive strategies in interactions with adults with Rett syndrome. Specific questions were whether the use of responsive strategies varied, (a) in interactions with different participants, (b) when the communication partner also used aided AAC modeling, and (c) whether the rate of confirmations and expansions differed between the participant’s digitized and non-digitized contributions to the interaction.

## Materials and methods

### Design

An exploratory case format focusing on the communication partner’s use of responsive strategies in dyadic interactions with three adults diagnosed with Rett syndrome was applied. The data were collected in an intervention study presented elsewhere (Wandin et al., 2021). During eight of 14 of the interactions, the communication partner used aided AAC modeling. Comparisons were made between the partner’s responsiveness under these two conditions, between participants and between responses to the participants’ digitized and non-digitized contributions to the interaction.

### Participants

The communication partner was recruited through a specialist center for assistive technology and AAC. The person was an AAC educational specialist with vast training in, and experience of, responsive strategies and aided AAC modeling, and extensive experience of working with individuals who use AAC to communicate.

Three women (27, 29, and 31 years of age) diagnosed with Rett syndrome were recruited as participants. They were all dependent on assistance for most of their daily activities. The Communication Matrix (Rowland, 2004) based on caregiver reports was used to assess the participants’ communicative skills before the study. The three participants were all assessed to be emerging symbol communicators, i.e., they used symbols to communicate sometimes or with support. They had used gaze-controlled computers but mainly for playing games and did not use them daily for communication. Participant 1 communicated through gaze direction and facial expressions and showed discomfort through vocalizations. She was reported to use yes/no signals that were consistent (blinking/moving head to the side) although the signals could be difficult to interpret for non-familiar communication partners. Participant 2 had subtle and infrequent body movements and the caregivers mostly relied on eye gaze and her alertness state to interpret her communication. When not content, she was reported to vocalize in a specific way. She used graphic symbols for “yes” and “no” to accept and reject options and confirm interpretations of her intention. Participant 3 used body movements and simple hand movements to communicate (reaching, pointing, and grasping) and also looked at graphic symbols to accept or reject objects. She was reported by her caregivers to have quick mood swings, and to often have a fleeting interest in activities.

The study obtained ethical approval from the Swedish Ethical Review Authority; Uppsala, Sweden (Dnr 2018/079) and ethical regulations and guidelines complied with Swedish law. Proxy consent was given by the legal guardians on behalf of the participants with Rett syndrome.

### Setting

All sessions took place individually at a specialist center for assistive technology and AAC in a room with few competing stimuli. The communication partner and the participants were seated at a table while interacting. The participant’s caregiver or carer was present in the room but did not take part in the interaction.

### Measures

The Responsive Augmentative and Alternative Communication Style Scale (RAACS) (Broberg et al., 2012), was used to assess the communicative partner’s responsive strategies from video-recorded interactions. This tool was chosen as it was developed for aided interaction and because of its psychometric qualities. RAACS had an acceptable percentage agreement (Broberg et al., 2012) in one study, and a Kappa agreement of 0.96, *p* < 0.001 was reported in a study by Stockwell et al. (2019). RAACS consists of 10 items covering responsive and scaffolding partner strategies (see Table 1). Lindberger (2020) assessed RAACS for interaction with pre-symbolic individuals. Inter-rater reliability was found to be poor for this group and adjustments were therefore suggested that increased the inter-rater reliability.

**Table 1:** Description of the RAACS items used in the current study.

Item 1	Is attentive to and confirms	The communication partner is attentive to and confirms the individual’s communication, for example by imitating, commenting, or labeling the individual’s physical and communicative actions.
Item 2	Adjusts physically	The communication partner adjusts physically to the individual by for example being close or turning to the individual.
Item 3	Gives space	The communication partner gives the individual space to communicate by, for example maintaining an easy pace and giving the individual enough time to communicate.
Item 4	Clarifies communication	The communication partner clarifies his or her own communication by, for example emphasising important words, using uncomplicated language, making use of objects or AAC present in the physical environment.
Item 5	Follows focus	The communication partner communicates according to the individual’s focus of interest or conversational topic by, for example observing and following in any distraction.
Item 6	Expands	The communication partner expands on the individual’s communication by, for example putting the individual’s communication into words by speaking orally or using a communication aid, or repeating and developing the content of the individual’s communication.
Item 7	Adapts^*^	The communication partner adapts to the individual: the overall impression of the communication partner’s ability to adapt to the individual’s actions and communication by, for example adapting to the individual’s pace.
Item 8	Is engaged^*^	The communication partner is emotionally responsive and shows warmth towards the individual for example by actively seeking eye contact, and is actively focused on the individual and on the mutual activity.
Item 9	Adjusts to communicative level	The communication partner adjusts to the communicative level of the individual by using communicative actions on the same or slightly above the communicative level of the individual.

The description is based on (Broberg et al., 2012). RAACS, version 3, can be downloaded from https://alfresco.vgregion.se/alfresco/service/vgr/storage/node/content/workspace/SpacesStore/81b1f634-c77d-4474-a8f9-a7c117fa9127/RAACS%20Version%203_130108.pdf?a=false&guest=true. The descriptions above are examples from the manual. In this table “child” is changed for “individual” and “parent” for “communication partner.”

* *Originally one item.*

Items 1–6 are rated minute-by-minute on a scale between 0 and 2 with “0” meaning that the strategy is not observed at all, “1” that the strategy is observed to occur occasionally or partly, and “2” that it occurs consistently during each observed minute. The average of the 10 min is then computed. For items 7–9, the communication partner’s use of the strategies is rated as occurring “never,” “sometimes,” or “often” during the whole 10-min clip (scale between 1 and 3). Observations and reflections regarding the interaction and/or coding process were also noted, such as “the communication partner is not attentive to and does not confirm breathing irregularities” or “the communication partner is waiting expectantly for much of the minute.” In the current study, the following adaptations were made:

- The original global item 8, “adapting and being engaged” was separated into two items to enhance reliability as suggested by Lindberger (2020). These descriptions were also used in the current study.
- The original item 7, “uses AAC” was excluded because the communication partner’s use of the gaze-controlled device was pre-determined for each session. Item 7 “uses AAC” in the original version, also includes pointing at objects, using signs, and offering choices. These behaviors were deemed to be reflected by item 4, “clarifies.” The maximum total score in the version used in the current study was therefore 21. All scores were noted in the RAACS protocol.
- In addition to RAACS, each confirmation from the communication partner following a digitized contribution (spoken using the gaze-controlled device) or a non-digitized contribution was recorded.
- Additionally, each expansion by the communication partner on digitized and non-digitized contributions from the participants was recorded.

### Inter-rater reliability

The first author coded all the video-recorded interactions using RAACS. The inter-rater reliability was calculated for 22% of the clips (*N* = 10), randomly selected through a stratified random sampling (selected from each participant and study phase). An external coder coded the 10 clips in a random order without receiving information about which study phase the clips were extracted from. To assess inter-rater reliability between the two coders, a 2-way mixed model, single rater, and absolute agreement IntraClass Correlation (ICC) was performed item-for-item (Koo and Li, 2016). An ICC of fair to excellent (0.73–0.96) was found.

### Procedure

Before the study, the caregivers suggested activities that would be possible to do sitting at a table, and that they assumed that the trial participant would enjoy. The communication partner tried to engage the participants in the activity but also made changes within an activity or changed activity following the participant’s interest and focus of attention. Activities included book reading, games on a smart plate, and listening to and discussing music. Two interaction sessions per day took place, and between the sessions, the trial participant and their caretakers engaged in any preferred restorative activity (such as going for a walk). Each session lasted 13-35 (*M=24*) min depending on the interest of the participant. The first 10 min of the interactions were coded using RAACS.

Responsive strategies were used in all interactions and a gaze-controlled device (Tobii I12 + ^™^), with a vocabulary specifically designed for the study, was used during all interactions. The vocabulary consisted of core words, comments, feelings, actions, descriptions, and activity-specific pages. The interactions took place under two conditions that were the two first phases in an intervention study presented elsewhere (Wandin et al., 2021):

- During the first six sessions, the communication partner was instructed to use responsive partner strategies and to engage the participants in the interaction and activities. The gaze-controlled device was placed on the table, slightly to the side but within the field of vision of the participant and the gaze-control was accessible to them. The dwell time (the length of time the user needs to focus their gaze to activate a choice on the screen) was set to 1 s. The communication partner was instructed to respond to all aided communication from the participants but not to point at, or in other ways draw attention to the device.
- During the following eight sessions, in addition to using responsive strategies, the communication partner pointed at symbols on the gaze-controlled device approximately twice each minute while they were speaking. There were no specific target words. Moreover, an individually set dwell time (500–800 milliseconds) was used to optimize access for the participants.

Each session was recorded with two video cameras; a Panasonic HCx9201^™^ video camera was used for recording the setting and a Sony HC22E^™^ camera was used for recording the screen of the gaze-controlled device. The recording started when the participant and the communication partner were seated and ended once the activities were stopped.

### Analyses

Descriptive statistics were used to present the RAACS results. The Wilcoxon signed-rank test (Bridge and Sawilowsky, 1999) was conducted to study differences between means (i.e., between the two study phases, and between digitized and non-digitized contributions), and the Friedman test (Hazra and Gogtay, 2016) was conducted to study differences between three means (i.e., differences between the trial participants). The significance level was pre-set at *p* < 0.05. SPSS version 25.0 was used for all analyses.

## Results

The mean scores for each item across both study phases and trial participants ranged between 1.25 and 3.00 (Table 2). In terms of items 1–6, that were scored minute by minute, item 6, “expands,” had the numerically lowest mean score, and item 2, “adjusts physically,” had the highest.

**Table 2:** Mean RAACS score per item (*n* = 3): total, and for each study phase.

Item	Total M (*SD*)	No intervention M (*SD*)	Intervention M (*SD*)	*Z* (*p*)
Is attentive to and confirms^*^ Adjusts physically^*^	1.42 (*0.71*) 1.98 (*0.21*)	1.29 (*0.71*) 2 (*0*)	1.51 (*0.70*) 1.96 (*0.27*)	−4.766 *(0.001)* −2.271 *(0.02)*
Gives space^*^	1.43 (*0.63*)	1.39 (*0.63*)	1.45 (*0.63*)	−2.651 *(0.008)*
Clarifies communication^*^	1.68 (*0.51*)	1.62 (*0.52*)	1.73 (*0.49*)	−3.939 *(0.001)*
Follows focus^*^	1.63 (*0.56*)	1.51 (*0.59*	1.71 (*0.52*)	−3.788 *(0.001)*
Expands^*^	1.25 (*0.77*)	1.07 (*0.71*)	1.39 (*0.79*)	−5.875 *(0.001)*
Is engaged	3.00 (*0*)	3.00 (*0*)	3.00 (*0*)	ns
Adapts	2.55 (*0.50*)	2.50 (*0.51*)	2.57 (*0.50*)	ns
Adjusts to communicative level	2.36 (*0.49*)	2.39 (*0.50*)	2.35 (*0.48*)	ns

* *p < 0.05 (Wilcoxon signed-rank test).*

### Responsive strategies between participants

The RAACS score varied between the three participants, with most of the lower scores being found in interactions involving participant 3 for all items except item 2, “adjusts physically” (Table 3). For item 2 the scores were lower when the communication partner interacted with participant 1. The differences for items 2, “adjusts physically,” 3, “gives space,” 4, “clarifies communication,” 5, “follows focus,” and 6, “expands” were significant at least with *p* < 0.05.

**Table 3:** RAACS score across participants.

	Participant 1	Participant 2	Participant 3	χ^2^(2)^1^, (*p*)
	M (*SD*)	M (*SD*)	M (*SD*)	
Is attentive to and confirms	1.41 (*0.69*)	1.53 (*0.64*)	1.31 (*0.79*)	4.696 (0*.096*)
Adjusts physically^**^	1.93 (*0.35*)	2 (*0)*	2 (*0*)	12 (0*.002*)
Gives space^**^	1.56 (*0.59*)	1.49 (*0.58*)	1.24 (*0.66*)	16.933 (0*.001*)
Clarifies communication^**^	1.84 (*0.37*)	1.72 (*0.45*)	1.50 (*0.61*)	26.771 (0*.001*)
Follows focus^*^	1.57 (*0.60*)	1.75 (*0.45*)	1.56 (*0.60*)	8.667 (0*.01)*
Expands^*^	1.28 (*0.72*)	1.38 (*0.73*)	1.11 (*0.84*)	7.408 (0*.03*)
Is engaged	3.00 (*0*)	3.00 (*0*)	3.00 (*0*)	ns
Adapts	2.64 (*0.50*)	2.64 (*0.51*)	2.31 (*0.47*)	ns
Adjusts to communicative level	2.21 (*0.43*)	2.57 (*0.51*)	2.31 (*0.47*)	ns

* *p < 0.05 and*

** *p < 0.005 (Friedman test)*.

1 *Chi-square, (df).*

### Responsive strategies across the study phases

In an item-by-item comparison, the difference was statistically significant between all items 1–6 (is attentive to and confirms, adjusts physically, gives space, clarifies communication, follows focus, and expands). No statistically significant differences were found for the global items 7–9 (adapts, is engaged, and adjusts communication). The differences were largest between the scores in the no intervention and intervention conditions for item 1, “is attentive to and confirms” (0.22), item 5, “follows focus” (0.20), and item 6, “expands” (0.32). The total RAACS score was numerically higher during the intervention phase (M = 17.8, SD = 1.9) than during the no-intervention phase (M = 16.7, SD = 1.39). However, this difference fell short of statistical significance (Z = 1.894, *p* = 0.058).

### Confirmations and expansions on digitized vs. non-digitized contributions

**Figure 1:**
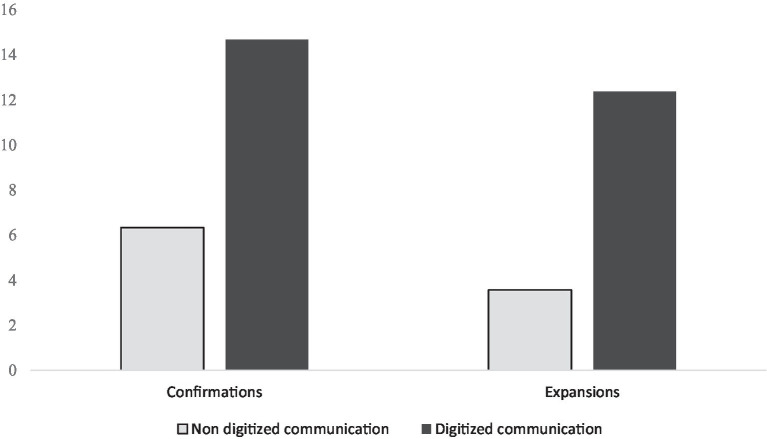
The rate of the communication partner’s confirmations to, and expansions on, the participants’ non digitized, and digitized communication. Both differences were significant, *p* < 0.005. For confirmations, Z = −3.597, for expansions, Z = −4.399.

The number of confirmations on the participants’ digitized contributions (M = 14.67, SD = 10.55, range 0–47) was higher than the number of confirmations on their non-digitized contributions (M = 6.33, SD = 4.75, range 0–20), (Z = −3.597, *p* < 0.005, see Figure 1). The number of expansions on the participants’ digitized contributions (M = 12.36, SD = 9.45, range 0–47) was also higher than on their non-digitized communication (M = 3.57, SD = 3.68, range = 0–16), (Z = −4.399, *p* < 0.005, See Figure 1).

## Discussion

The mean scores for all items that were coded minute by minute were at least 1.25. The communication partner thus used the targeted behaviors on average at least “sometimes” during the 10 coded minutes.

### Responsive strategies in interaction with different participants

The RAACS score differed between the interactions with the three participants, with the highest total score being in the interactions with trial participant 2 and the lowest being in interactions with trial participant 3. Thus, the results indicate that the use of responsive strategies is influenced by the person with whom the communication partner interacts. Models underpinning the practice of responsive strategies propose that the actions of both individuals in a dyad influence each other. However, to the best of our knowledge, the current study is the first observational study exploring whether the use of responsive strategies varies when a communication partner interacts with different non-speaking individuals. Earlier research on parents’ use of responsive strategies found that the type of disability correlated with the parents’ responsivity (Brooks-Gunn and Lewis, 1984). However, these findings are not entirely supported by more recent studies (Cress et al., 2013; Karaaslan and Mahoney, 2013). In the study by Van Keer et al. (2017), neither the age, gender, nor sensory impairment of children with significant cognitive and motor developmental delay was related to their parents’ use of responsive strategies. The current study indicates a need to go beyond diagnosis to identify factors related to the non-speaking individual that may influence the communication partner’s use of responsive strategies. Variety and rate of intentional communication and communication modes are examples of factors that have been reported to increase the communication partner’s responsivity (Yoder and Warren, 1999, 2001; Cress et al., 2013).

### Responsive strategies when using aided AAC modeling

The fact that the RAACS score was higher during the intervention phase was slightly unexpected as using aided AAC modeling places more demands on the communication partner’s ability to shift their focus of attention. Examination of the results item per item showed that the largest increase was for item 6, “expands” followed by item 1, “is attentive to and confirms.” As the number of digitized contributions was higher during the intervention phase than in the no-intervention phase for all three participants, this difference may be a contributing factor. This result is in line with earlier findings that intentional communicative behavior as well as vocal communication may elicit the communication partner’s use of responsive strategies (Yoder and Warren, 2001). Gaze-controlled devices appear to provide individuals with Rett syndrome with an accessible form of expressive communication. This may have, for example, influenced the time that the communication partner needed to give the individual “enough time to communicate” as described in RAACS item 3, “gives space.” The participants’ increased use of the gaze-controlled device may thus have contributed to the higher RAACS score during the intervention phase. An individualized dwell time was also introduced in the intervention phase.

### Confirmations and expansions on digitized and non-digitized contributions

The number of the communication partner’s confirmations and expansions on the participants’ digitized contributions was higher than on their non-digitized contributions. For individuals with a limited repertoire of expressive communication, this is of great importance for participation. In Medeiros and Cress (2016) the responsivity of mothers of children with complex communication needs did not differ between interactions when a communication device was used and those when one was not used. In the current study, the response rate was significantly higher when participants communicated through the gaze-controlled device. In the study by Medeiros et al., the communication device was a single message device, in contrast to a gaze-controlled device with a larger vocabulary. All the participants were able to activate the device, which requires a certain level of motor control. In the current study, only one of the three participants was able to use her hands to access communication devices. These differences between participants and communication devices included in the studies may explain the varying results. In the study by Shalev and Hetzroni (2020), school staff were more responsive in interactions with students who used speech than with those who did not communicate through speech. The authors hypothesize that speech, even when limited, may be easier to perceive and relate to a potential intent than non-verbal communication. The digitized contributions in this study may similarly have been easier to perceive and interpret for the communication partner than the participants’ non-verbal communication. The strategy to expand on communication is aimed at facilitating language development (Clarke et al., 2017; Soto et al., 2020) and more expansions may thus stimulate language development.

### Responsive strategies and directiveness

As part of the study procedure the communication partner engaged the participants in an activity and used aided AAC modeling approximately twice per minute during the intervention. This may have influenced the communication partner to be more directive, e.g., to be less inclined to “observe and follow in the case of any distraction” which is included in item 5, “follow focus.” It is possible that the communication partner would have been even more responsive in a less achievement-focused activity (Cress et al., 2013). However, there are several situations in everyday life in the home, at school, or at adult daycare centers when tasks must be performed, often within a limited time. Individuals with Rett syndrome spend a large part of the day on daily care and health care activities according to a study by Sernheim et al. (2019). It may therefore be useful to assess the use of responsive strategies in these situations and provide training to increase the communication partner’s use of responsive strategies when needed.

### Limitations

There are numerous limitations to this study. First, the current study was an explorative case study, including one person interacting with three individuals. Since it is a case study, the results cannot be generalized to other communication partners, participants, and conditions, nor can any causal inferences be drawn. Second, RAACS was originally developed to measure parents’ responsive communication styles. The items and operationalizations do not, at face value, seem specific to parenting. Nonetheless, there may be differences in responsivity and the RAACS profile due to the different roles. For example, the communication partner in the current study was an AAC educational specialist. Therapists and educators may be more focused on teaching and promoting development than parents, while parents may or may not be more focused on enhancing existing communication. Third, there may also be differences in how therapists and parents interpret the communicative behavior of individuals with Rett syndrome, for example in terms of communicative function (if any) of the behavior (Julien et al., 2014). However, this should only affect the results and not the scoring procedure, and RAACS thus appears to be suitable for the aim of the current study, i.e., to explore and describe a trained communication partner’s use of responsive partner strategies in aided interaction with adults with Rett syndrome.

## Conclusion

Although responsivity is a broad construct, it is often reported as a composite score. The detailed assessment revealed that the communication partner’s use of responsive and scaffolding strategies is not a fixed construct but varies in interactions with different non-speaking persons as well as in different contexts. RAACS and the resulting RAACS profile may be used to assess a communication partner’s interaction patterns in a dyad, providing clinically valuable knowledge. Knowledge of the interaction patterns for the individual dyad could be used when planning intervention aimed at increasing the communication partner’s use of responsive strategies. The number of responses and the use of responsive strategies were higher when the participants used a gaze-controlled device. A higher rate of responses indirectly increases the number of initiating turns of individuals with Rett syndrome.

## Data availability statement

The raw data supporting the conclusions of this article will be made available by the authors, without undue reservation.

## Ethics statement

The studies involving human participants were reviewed and approved by the Swedish Ethical Review Authority; Uppsala, Sweden. Written informed consent to participate in this study was provided by the participants’ legal guardian/next of kin.

## Author contributions

All authors designed the work. The first author collected and analyzed all data. All authors contributed to the article and approved the submitted version.

## Funding

This study was supported by the Swedish National Center for Rett syndrome and Related Disorders, Region Jämtland Härjedalen, Sweden, the Department of Public Health and Caring Sciences, Uppsala University and the Linnéa och Josef Carlsson Foundation, and Inga Henriksson Memorial.

## Conflict of interest

The authors declare that the research was conducted in the absence of any commercial or financial relationships that could be construed as a potential conflict of interest.

## Publisher’s note

All claims expressed in this article are solely those of the authors and do not necessarily represent those of their affiliated organizations, or those of the publisher, the editors and the reviewers. Any product that may be evaluated in this article, or claim that may be made by its manufacturer, is not guaranteed or endorsed by the publisher.
